# Prevalence of Malaria and Leptospirosis Co-Infection among Febrile Patients: A Systematic Review and Meta-Analysis

**DOI:** 10.3390/tropicalmed6030122

**Published:** 2021-07-03

**Authors:** Polrat Wilairatana, Wanida Mala, Pongruj Rattaprasert, Kwuntida Uthaisar Kotepui, Manas Kotepui

**Affiliations:** 1 Department of Clinical Tropical Medicine, Faculty of Tropical Medicine, Mahidol University, Bangkok 10400, Thailand; polrat.wil@mahidol.ac.th; 2 Medical Technology, School of Allied Health Sciences, Walailak University, Tha Sala, Nakhon Si Thammarat 80161, Thailand; wanida.ma@wu.ac.th (W.M.); kwuntida.ut@wu.ac.th (K.U.K.); 3 Department of Protozoology, Faculty of Tropical Medicine, Mahidol University, Bangkok 10400, Thailand; pongruj.rat@mahidol.ac.th

**Keywords:** malaria, leptospirosis, co-infection

## Abstract

Malaria and leptospirosis are important cosmopolitan infections that have emerged with overlapping geographic distribution, especially in tropical and subtropical regions. Therefore, co-infection with malaria and leptospirosis may occur in overlapping areas. The present study aimed to quantify the prevalence of malaria and leptospirosis co-infection among febrile patients. The association between malaria and leptospirosis infections was also investigated. Relevant studies that had reported malaria and leptospirosis co-infection were identified from PubMed, Scopus, and Web of Science. The risk of bias of the studies was assessed using the Joanna Briggs Institute (JBI) Critical Appraisal Tool. The pooled prevalence of malaria and leptospirosis co-infections among febrile patients and the pooled prevalence of leptospirosis infection among malaria patients were estimated using random effect models. The association between malaria and leptospirosis infection among febrile patients was estimated using random effect models. The outcomes of each study were shown in a forest plot in point estimate and 95% confidence interval (CI). Heterogeneity among the included studies was assessed using Cochran’s Q and quantified using I-squared statistics. For leptospirosis, subgroup analyses of countries, diagnostic tests, and participants’ age groups were performed to specify prevalence in each subgroup. Publication bias was assessed by funnel-plot visualization. Of the 2370 articles identified from the databases, 15 studies met the eligibility criteria and were included for qualitative and quantitative syntheses. Most of the included studies were conducted in India (5/15, 33.3%), Thailand (3/15, 20%), and Cambodia (2/15, 13.3%). Most of the enrolled cases were febrile patients (5838 cases) and malaria-positive patients (421 cases). The meta-analysis showed that the pooled prevalence of malaria and leptospirosis co-infection (86 cases) among febrile patients was 1% (95% CI: 1–2%, I^2^: 83.3%), while the pooled prevalence of leptospirosis infection (186 cases) among malaria patients was 13% (95% CI: 9–18%, I^2^: 90.3%). The meta-analysis showed that malaria and leptospirosis co-infections occurred by chance (p: 0.434, OR: 1.4, 95% CI: 0.6–3.28, I^2^: 85.2%). The prevalence of malaria in leptospirosis co-infection among febrile patients in the included studies was low. Co-infection was likely to occur by chance. However, as clinical symptoms of leptospirosis patients were non-specific and not distinguishable from symptoms of malaria patients, clinicians caring for febrile patients in an area where those two diseases are endemic should maintain a high index of suspicion for both diseases and whether mono-infections or co-infections are likely. Recognition of this co-infection may play an important role in reducing disease severity and treatment duration.

## 1. Introduction

Malaria in humans is caused by one of six *Plasmodium* spp.: *P. falciparum*, *P. vivax*, *P. malariae*, *P. ovale curtisi*, *P. ovale wallikeri*, and *P. knowlesi* [1]. Recent epidemiological studies have shown that *P. cynomolgi* might be a cause of malaria in humans in Cambodia [2], Thailand [3], and Malaysia [4,5,6]. The World Health Organization (WHO) estimated 229 million malaria cases were reported in 2019, out of which African countries accounted for about 94% of cases [7]. Symptoms of malaria ranged from asymptomatic and uncomplicated to malaria with severe complications [8]. If left untreated or with treatment delay, severe complications of malaria could occur. These were mostly caused by *P. falciparum* infection; however, a lesser proportion of severe malaria could also be caused by other *Plasmodium* spp. [9,10,11,12,13]. As the patients with uncomplicated malaria presented with non-specific signs or symptoms, such as fever, general malaise, headache, arthralgia, or myalgia, the clinical diagnosis of malaria could be confounded by other acute undifferentiated febrile illness (AUFI), such as enteric fever, dengue fever, rickettsiosis, Japanese encephalitis, and leptospirosis, which share similar clinical presentations [14,15].

Leptospirosis is one of the most important zoonotic diseases caused by pathogenic species of the spirochete bacteria *Leptospira* [16,17]. This disease is considered a neglected and re-emerging disease of global public health significance, which causes high mortality and morbidity in both humans and animals [18,19]. Nowadays, outbreaks of leptospirosis occur in tropical countries, particularly India, Malaysia, and Brazil [17]. It is also a predominant cause of febrile illness in South America [17]. Leptospirosis cases were found to increase due to heavy rainfall, flooding, and poor sanitation, which frequently occur in urban slum areas [20,21]. Previous studies demonstrated the incidence of leptospirosis increasing and being widespread in Italy, Pakistan, Japan, Nicaragua, the Philippines, and Sri Lanka [17,18]. At least 1 million leptospirosis cases have been reported worldwide, with nearly 60,000 deaths per year [22]. The initial non-specific symptoms presenting as febrile illness can result in misdiagnosis with other diseases such as malaria, dengue, and Zika virus infections [18,23]. This can result in an increase in fatalities in the severe stage of leptospirosis (Weil’s disease) [18]. Therefore, the early diagnosis of leptospirosis infection can decrease the severity of disease. The clinical manifestations of leptospirosis range from asymptomatic or initially presenting as a flu-like febrile illness, through mild to severe infections [16]. Severe cases can develop into Weil’s disease, characterized by multi-organ failure and complications, including jaundice, pulmonary hemorrhage, and acute renal failure [16,19]. Transmission can occur by direct contact with an infected animal or indirect contact with the environment through open wounds, abrasions, and mucous membranes such as water and soil contaminated with the urine of infected animals [16]. However, the most frequent exposure route of infection in humans is indirect contact. Animal reservoirs of leptospirosis include rodents (particularly rats), pigs, horses, cattle, dogs, and other wild animals [19,23]. These bacteria persist and accumulate in their reservoir’s kidneys before being excreted in urine [24,25]. Risk groups for infection involve people whose occupations require interaction with an infected animal, agriculture (e.g., farmers), veterinarians, and persons in contact with water [25].

Malaria and leptospirosis are important cosmopolitan infections that have emerged with overlapping geographic distribution, especially in tropical and subtropical regions [26]. Therefore, co-infection with malaria and leptospirosis may occur in overlapping areas. The present study aimed to quantify the prevalence of malaria and leptospirosis co-infection among febrile patients. The association between malaria and leptospirosis infections was also investigated.

## 2. Methods

### 2.1. Protocol and Registration

The protocol of this systematic review was registered at PROSPERO with ID CRD42021255898. Reports of the systematic review followed the PRISMA 2020 statement [27].

### 2.2. Information Sources

Potentially relevant articles were searched in PubMed, Scopus, and Web of Science by using keyword combinations specific for malaria and leptospirosis, as provided in Table S1. The searches were not limited by language or publication year. Additional searches from referent lists of the included studies or review articles and searches in Google Scholar were performed to avoid missing studies related to this study.

### 2.3. Eligibility Criteria

All types of study designs that reported malaria and leptospirosis were considered. Studies were selected according to the following eligibility (inclusion/exclusion) criteria: (1) Cross-sectional studies, longitudinal studies, case-control studies, cohort studies, and observational studies were included. The inclusion of all types of studies allowed us to maximize the number of included studies to represent the pooled prevalence of co-infection globally and (2) only human studies with malaria and leptospirosis infections by laboratory diagnosis, such as microscopic diagnosis, culture, molecular diagnosis, rapid diagnostic test (RDT), and serology, were included. The following studies were excluded: diagnosis of malaria and leptospirosis infection by clinical diagnosis only (patient symptoms and physical examination), animal studies, in vitro studies, assay performance, review articles, case reports, and case series. The participant/population (P), outcome of interest (I), and contexts (Co) were applied to the key question.

### 2.4. Study Selection

Study selection was based on the eligibility criteria. Articles were retrieved from the databases using the search strategy. All articles were imported into Endnote software for management. All studies were reviewed by two independent authors (MK and WM). First, duplicates were screened and removed. Second, the titles and abstracts of the articles were reviewed. Unrelated articles were excluded and then the remaining articles were examined for full texts. Studies that met the eligibility criteria were included and those that did not were excluded, with the explained reasons. For any discrepancies between the two authors during study selection, another author (PW) served as a third author to create consensus.

### 2.5. Data Extraction

The two authors (MK and WM) extracted data from each included study to the pilot Excel datasheet before data analysis. The following data were extracted: name of the first author, year of publication, country, year study conducted, study design, characteristics of participants enrolled, age, gender, number of patients with co-infection, number of patients with malaria, number of patients with leptospirosis, diagnostic test(s) for malaria, and diagnostic test(s) for leptospirosis. The data were cross-checked by another author (PW) to assure the accuracy of the method.

### 2.6. Quality of the Included Studies

The risk of bias in the studies was assessed independently by two reviewers (MK and WM) according to the Joanna Briggs Institute (JBI) Critical Appraisal Tools for cross-sectional study [28]. Any disagreement between the two reviewers was resolved by consensus by a third author (PW). The key aspects of the JBI Critical Appraisal Tools for the cross-sectional study are the following: (1) clearly defined criteria for inclusion in the sample; (2) description of study subjects and setting; (3) the exposure was measured validly and reliably; (4) objective and standard criteria used for measurement of the condition; (5) identification of confounding factors; (6) strategies to deal with confounding factors; (7) outcomes were measured validly and reliably; and (8) use of appropriate statistical analysis. For quality assessment, “High”, “Moderate”, or “Low” quality was rated for any studies given over 7 scores, 4–6 scores, and less than 4 scores, respectively.

### 2.7. Data Synthesis and Statistical Analysis

The data extracted from all of the included studies were narratively synthesized to provide a qualitative account of the data extracted from the included studies. The qualitative syntheses involved an explanation of the characteristics of the included studies, including study design, participants, study location, age, number of patients with co-infections, number of patients with malaria, number of patients with leptospirosis, and diagnostic tests for both malaria and leptospirosis. The quantitative synthesis involved the use of statistical analysis to pool the outcome. The first outcome of this study was the pooled prevalence of malaria and leptospirosis co-infections among febrile patients. The secondary outcome was the pooled prevalence of leptospirosis infection among malaria patients. The tertiary outcome was the pooled odds of malaria and leptospirosis co-infections among febrile patients. All outcomes were estimated using the random effect models and assumed that heterogeneity existed among the included studies. The outcomes of each study were shown in the forest plot in the point estimate and their 95% confidence interval (CI). The summarized outcome of interest was also shown in the forest plots. The heterogeneity among the included studies was assessed using Cochran’s Q and quantified using I-squared statistics. In the presence of substantial heterogeneity, the outcomes were pooled using the random effect model. For leptospirosis, the subgroup analyses of countries, diagnostic tests, and participants’ age groups were performed to specify the prevalence in each subgroup. Publication bias was assessed by visualizing a funnel plot. The meta-analysis was conducted using Stata ver. 14 (StataCorp, College Station, TX, USA).

## 3. Results

### 3.1. Search Results

**Figure 1:**
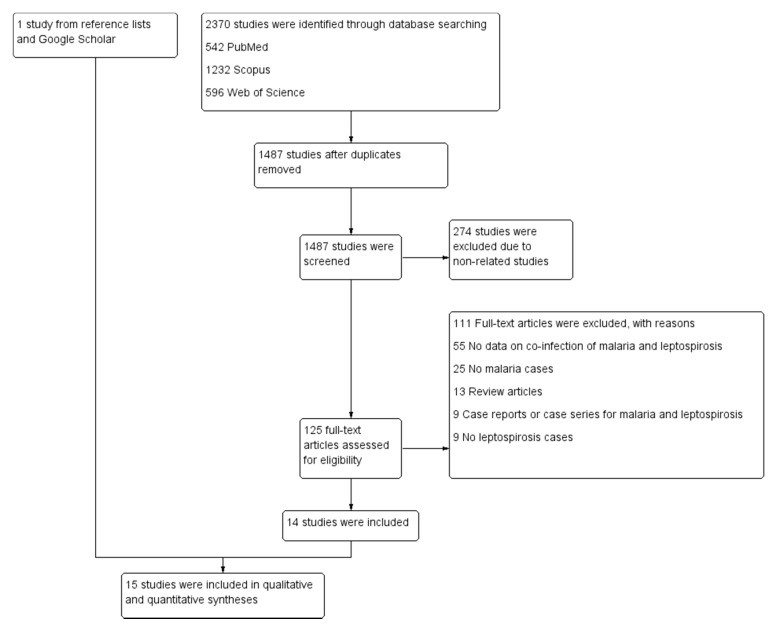
Study flow diagram. Study selection process.

Overall, 2370 articles were retrieved from three databases: 542 from PubMed, 1232 from Scopus, and 596 from the Web of Science. After duplicates were removed, 1487 studies were screened for titles and abstracts. After 247 non-relevant articles were excluded, 125 articles were examined for full texts. Of the 125 articles examined for full text, 111 articles were excluded for the following reasons: 55 had no data on co-infection of malaria and leptospirosis, 25 had no malaria cases, 13 were review articles, 9 were case reports or case series for malaria and leptospirosis, and 9 had no leptospirosis cases. Fourteen articles [26,29,30,31,32,33,34,35,36,37,38,39,40,41] met the eligibility criteria and were included in the qualitative synthesis. Additional searches on reference lists and Google Scholar found one article [42]. Finally, 15 articles [26,29,30,31,32,33,34,35,36,37,38,39,40,41,42] were included in the qualitative and quantitative syntheses (Figure 1).

### 3.2. Characteristics of the Included Studies

Fifteen studies included in the present study were prospective observational studies (7/15, 46.7%) [30,32,33,34,37,41], cross-sectional studies (6/15, 40%) [29,31,36,39,41,42], and retrospective observational studies (2/15, 13.3%) [37,42]. All studies were published between the years 2003–2021. Most of the included studies were conducted in India (5/15, 33.3%) [32,34,36,37,41], Thailand (3/15, 20%) [30,38,40], Cambodia (2/15, 13.3%) [33,35], Bangladesh [39], Jamaica [31], Malaysia [26], Tanzania [29], and Venezuela [42]. Most studies enrolled febrile patients (5838 cases) (10/15, 66.7%) [29,30,31,33,34,36,37,39,40,41], malaria-positive patients (421 cases) [32,38,42], and one study enrolled both febrile and non-febrile individuals (1193 cases) [35]. Most of the included studies enrolled adult patients (7/15, 46.7%) [26,30,34,37,38,41,42], all age groups (5/15, 33.3%) [31,33,35,36,39], and age not specified, by Mandage et al. [32]. For malaria diagnosis, most of the included studies used a gold standard “microscopy” alone (8/15, 53.3%) [26,29,30,33,36,38,41,42], microscopy/RDT/PCR (3/15, 20%) [32,35,39], microscopy/RDT [34] ELISA [31] microscopic agglutination test (MAT) [40], and RDT [37]. For leptospirosis diagnosis, most of the included studies used ELISA (7/15, 46.7%) [31,34,36,39,41,42], ELISA/MAT (4/15, 26.7%) [29,30,33,40], PCR [32,35], IFA [38], MAT alone [37], and MAT/PCR [26]. All of the characteristics of the included studies are shown in Table 1.

### 3.3. Quality of the Included Studies

The quality of the included studies was assessed using the checklist (see Supplementary Material S1) for analytical cross-sectional studies developed by the Joanna Briggs Institute [28]. Most of the included studies were moderate-quality studies [31,32,34,36,37,38,39,41,42], while six studies were high-quality [26,29,30,33,35,40].

### 3.4. Prevalence of Malaria and Leptospirosis Co-Infection among Febrile Patients

**Figure 2:**
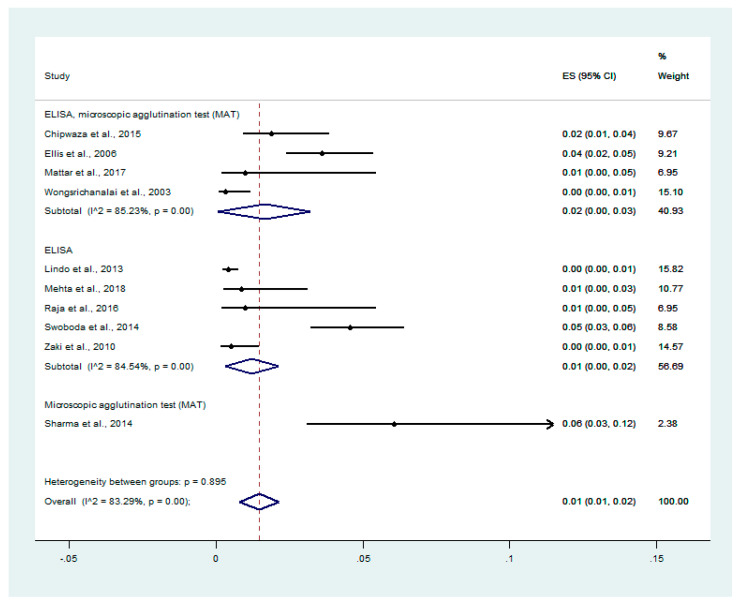
Prevalence of malaria and leptospirosis co-infection among febrile patients by diagnostic tests. % Weighted: the impact proportion of each study to the pooled effect; black dot symbol on black horizontal line: point estimate for each study; black horizontal line: CI, white diamond symbol: pooled prevalence; CI: confidence interval; ES: effect size (prevalence).

The prevalence of malaria and leptospirosis co-infection among febrile patients was estimated from 10 studies that enrolled 5838 febrile patients [29,30,31,33,34,36,37,39,40,41]. The results showed that the pooled prevalences of malaria and leptospirosis co-infection among febrile patients in studies using ELISA/MAT, ELISA alone, and MAT alone for diagnosed leptospirosis were 2% (95% CI: 0–3%, I^2^: 85.2%), 1% (95% CI: 0–2%, I^2^: 84.5%), and 6% (95% CI: 3–12%), respectively (Figure 2).

**Figure 3:**
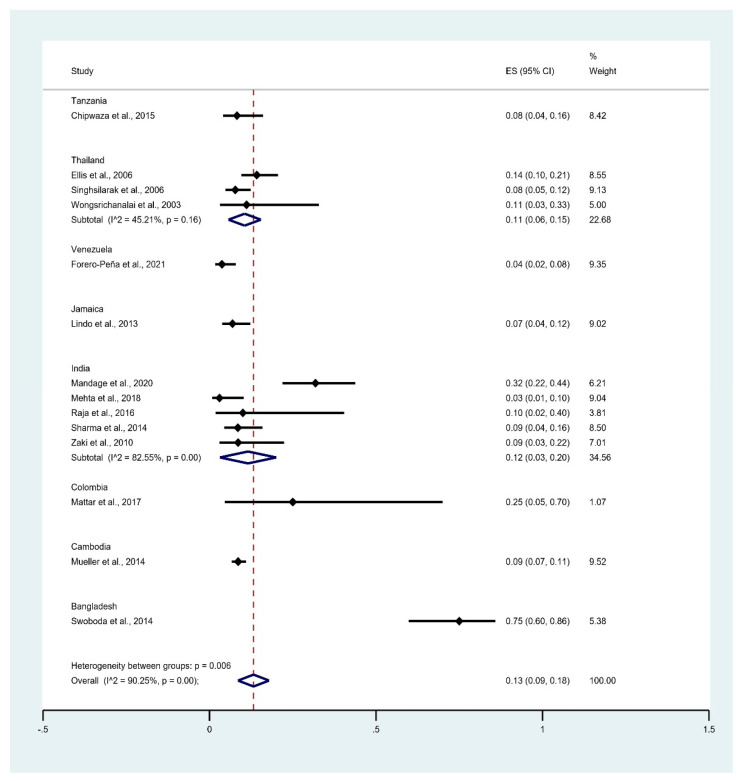
Prevalence of malaria and leptospirosis co-infection among febrile patients by country. % Weighted: the impact proportion of each study to the pooled effect; black dot symbol on black horizontal line: point estimate for each study; black horizontal line: CI, white diamond symbol: pooled prevalence; CI: confidence interval; ES: effect size (prevalence).

The subgroup of countries showed that the pooled prevalence of malaria and leptospirosis co-infection among febrile patients was 5% in Bangladesh (95% CI: 3–6%), 2% in Tanzania (95% CI: 1–4%), 1% in Thailand (95% CI: 0–1%, I^2^: 98.1%), 0% in Jamaica (95% CI: 0–1%), 1% in Colombia (95% CI: 0–5%), and 1% in India (95% CI: 0–2%, I^2^: 59%). Overall, the pooled prevalence of malaria and leptospirosis co-infection (86 cases) among febrile patients was 1% (95% CI: 1–2%, I^2^: 83.3%) (Figure 3).

### 3.5. Prevalence of Leptospirosis Infection among Malaria Patients

**Figure 4:**
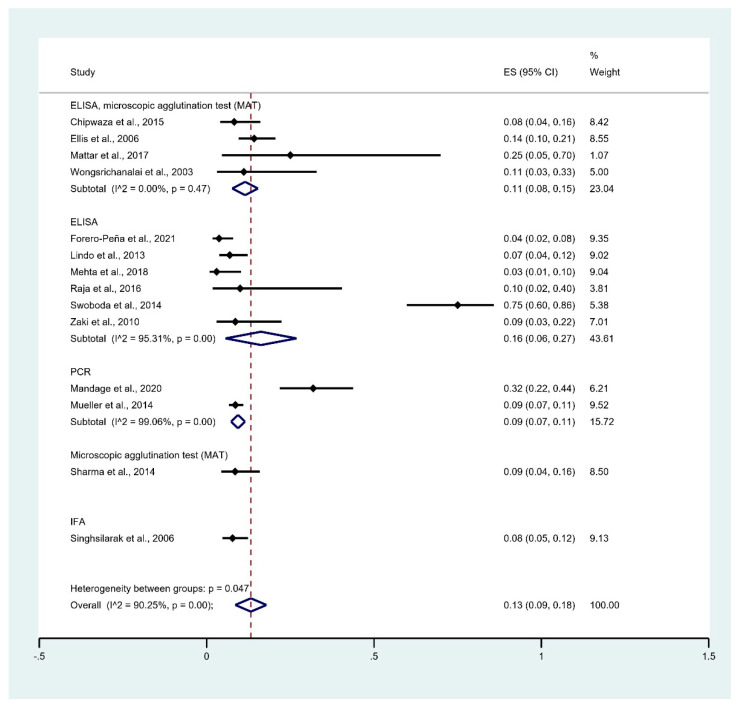
Prevalence of leptospirosis infection among malaria patients by diagnostic test. % Weighted: the impact proportion of each study to the pooled effect; black dot symbol on black horizontal line: point estimate for each study; black horizontal line: CI, white diamond symbol: pooled prevalence; CI: confidence interval; ES: effect size (prevalence).

The prevalence of leptospirosis infection among malaria patients was estimated from 14 studies that enrolled 1750 malaria patients [29,30,31,32,33,34,35,36,37,38,39,40,41,42]. The results showed that the pooled prevalence of leptospirosis infection among malaria patients in studies using ELISA/MAT, ELISA alone, PCR, PCR, MAT alone, and IFA alone for diagnosing leptospirosis were 11% (95% CI: 8–15%, I^2^: 0%), 9% (95% CI: 7–11%, I^2^: 99.1%), 9% (95% CI: 4–16%), 6% (95% CI: 3–12%), and 8% (95% CI: 5–12%), respectively (Figure 4).

**Figure 5:**
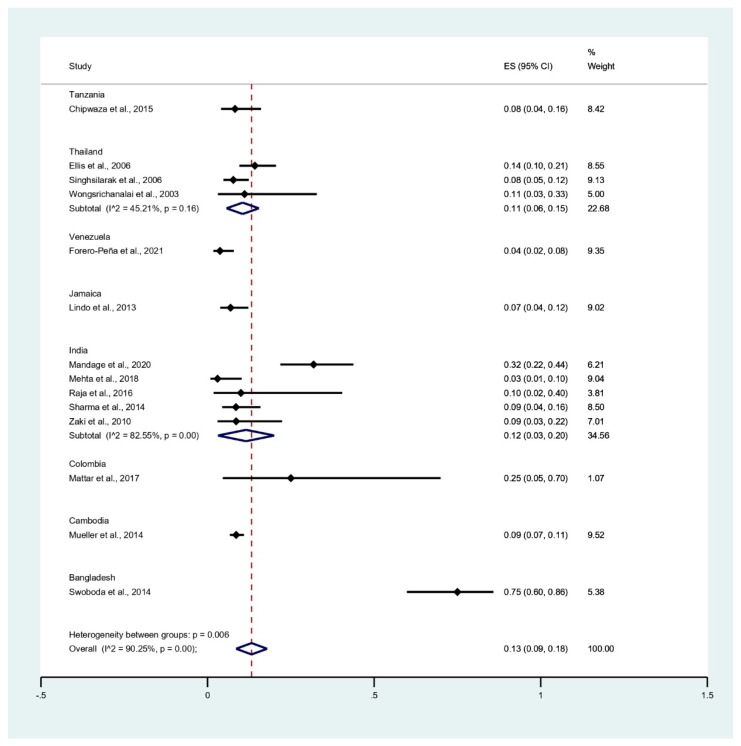
Prevalence of leptospirosis infection among malaria patients by country. % Weighted: the impact proportion of each study to the pooled effect; black dot symbol on black horizontal line: point estimate for each study; black horizontal line: CI, white diamond symbol: pooled prevalence; CI: confidence interval; ES: effect size (prevalence).

The subgroup of countries showed that the pooled prevalence of leptospirosis infection among malaria patients was 75% in Bangladesh (95% CI: 60–86%), 25% in Colombia (95% CI: 5–70%), 12% in India (95% CI: 3–20%, I^2^: 82.6%), 11% in Thailand (95% CI: 6–15%, I^2^: 45.2%), 9% in Cambodia (95% CI: 7–11%), 8% in Tanzania (95% CI: 4–16%), 7% in Jamaica (95% CI: 4–12%), and 4% in Venezuela (95% CI: 2–8%) (Figure 5).

**Figure 6:**
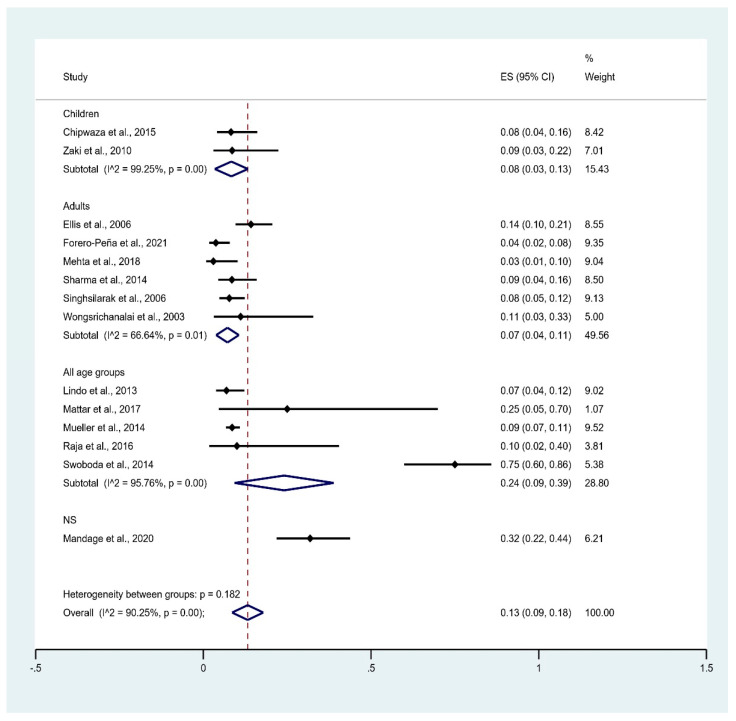
Prevalence of leptospirosis infection among malaria patients by age group. % Weighted: the impact proportion of each study to the pooled effect; black dot symbol on black horizontal line: point estimate for each study; black horizontal line: CI, white diamond symbol: pooled prevalence; CI: confidence interval; ES: effect size (prevalence).

The subgroup analysis of age groups showed that the pooled prevalence of leptospirosis infection among malaria patients was 8% in children (95% CI: 3–13%, I^2^: 99.3%), 7% in adults (95% CI: 4–11%, I^2^: 66.6%), and 24% in all age groups (95% CI: 9–39%, I^2^: 95.8%). Overall, the pooled prevalence of leptospirosis infection (186 cases) among malaria patients was 13% (95% CI: 9–18%, I^2^: 90.3%) (Figure 6).

### 3.6. Odds of Malaria and Leptospirosis Co-Infections

**Figure 7:**
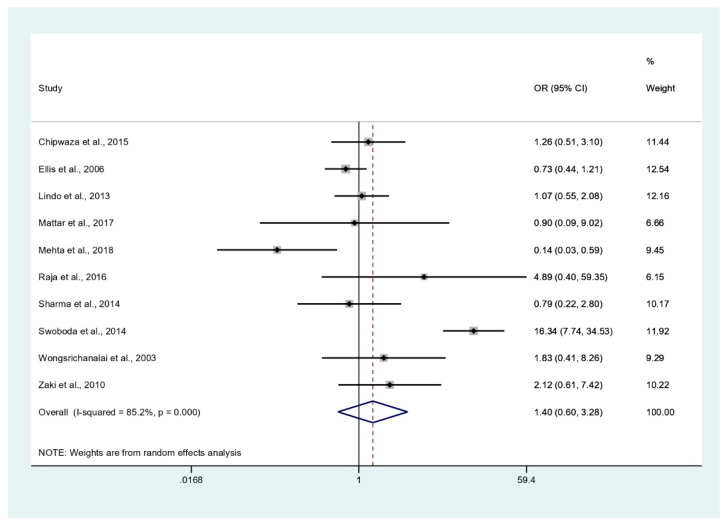
Odds of malaria and leptospirosis co-infections. % Weighted: the impact proportion of each study to the pooled effect; black dot symbol on black horizontal line: point estimate for each study; black horizontal line: CI, white diamond symbol: odds ratio; CI: confidence interval; ES: effect size (odds ratio).

The odds of malaria and leptospirosis co-infections (86 cases) among febrile patients (5838 cases) were estimated using the data of 10 studies [29,30,31,33,34,36,37,39,40,41]. The results of the individual study showed the lower odds of co-infection in one study conducted in India (OR: 0.14, 95% CI: 0.03–0.59) [34], while the higher odds of co-infection was demonstrated in one study conducted in Bangladesh (OR: 16.34, 95% CI: 7.74–34.5) [39]. Overall, the meta-analysis showed that malaria and leptospirosis co-infections occurred by chance (p: 0.434, OR: 1.4, 95% CI: 0.6–3.28, I2: 85.2%) (Figure 7).

### 3.7. Publication Bias

**Figure 8:**
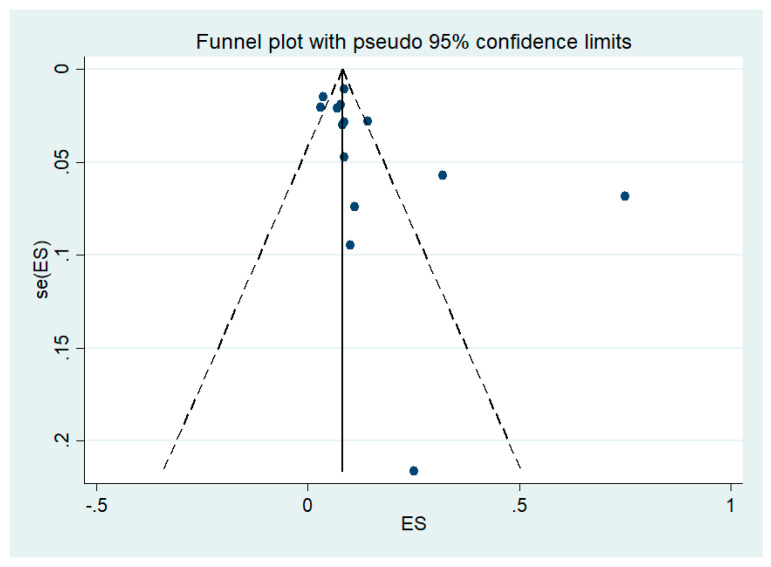
Funnel plot. ES: effect size (odds ratio), se: standard error.

Publication bias among studies that included analysis of the pooled prevalence of leptospirosis infection among malaria patients was performed using funnel plot, Egger’s test, and Contour enhanced funnel plot. The funnel plot between effect size (ES, pooled prevalence) and standard error of the ES (seES) showed the asymmetrical distribution of the outcomes of two studies (Figure 8).

**Figure 9:**
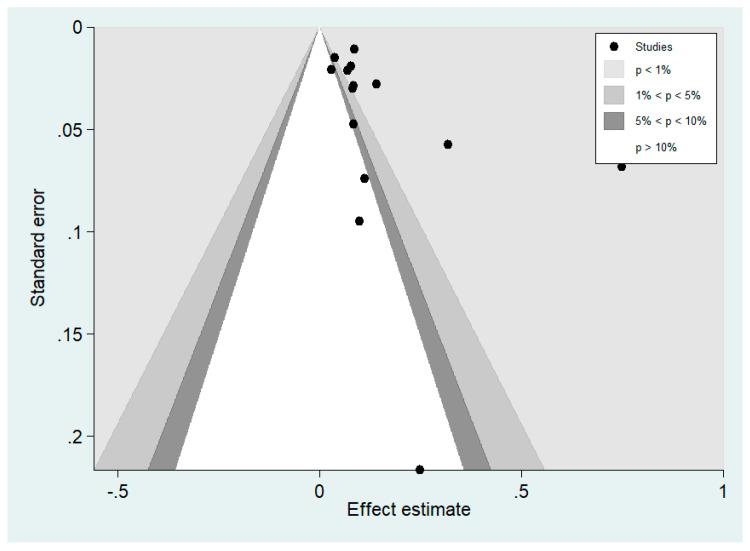
Contour-enhanced funnel plot.

The result of Egger’s test showed a non-significant small study effect (*p*: 0.07, coefficient: 2.75, standard error: 1.38, t; 1.99). The contour-enhanced funnel plot showed missing studies in non-significant areas (*p* > 0.01) indicating that the cause of funnel plot asymmetry may more likely be due to publication bias (Figure 9).

## 4. Discussion

The clinical signs and symptoms of uncomplicated malaria and leptospirosis were similar, making accurate clinical diagnosis difficult without laboratory confirmation. The meta-analysis showed that the overall pooled prevalence of malaria and leptospirosis co-infection among febrile patients was low (1%). However, the subgroup of countries showing highest co-infection was in Bangladesh (5%) and lower in Tanzania (2%), Thailand, Jamaica, Colombia, and India. In addition, the pooled prevalence of leptospirosis infection among malaria patients was high (13%). However, the heterogeneity of the prevalence was subsided by the subgroup of countries that showed the highest prevalence in Bangladesh (75%) and lower in Colombia (25%), India (12%), Thailand (11%), Cambodia (9%), Tanzania (8%), Jamaica (7%), and Venezuela (4%). The high prevalence of leptospirosis infection among malaria patients in Bangladesh could be explained by the seropositivity for leptospirosis being stable throughout seasonality, while malaria had a peak in the rainy season when conditions for the vector seem to be favorable. In addition, high proportions of asymptomatic malaria-positive adults were identified in this country, indicating a greater probability for semi-immunity by increasing age [39]. Moreover, the diagnostic tool for leptospirosis diagnosis in the study in Bangladesh was ELISA, which is not the standard method for leptospirosis diagnosis. However, IgM seropositivity by ELISA can indicate recent *Leptospira* infection [39]. While the gold standard for malaria diagnosis required microscopic examination of malaria parasites, the gold standard for leptospirosis diagnosis depended on serological tests; the microscopic agglutination test (MAT) had high sensitivity in the early stage of leptospirosis infection [19]. The titer value of MAT ≥ 400 or a four-fold rise in antibody titer between acute and convalescent sera is considered positive for leptospirosis infection [26,43]. In addition, enzyme-linked immunosorbent assay (ELISA), immunofluorescence assay (IFA) and indirect hemagglutination assay (IHA), and molecular techniques, such as nested polymerase chain reaction (PCR) and real-time PCR, can be used for diagnosed leptospirosis [18]. ELISA is widely used to detect the presence of specific IgM and IgG antibodies from patient sera. However, paired sera testing by ELISA is required for confirmation by MAT assay [43]. The IFA assay is based on the recognition of leptospiral surface protein by specific antibodies [25]. This assay is rapid and requires observation under a fluorescence microscope. In addition, the molecular technique can aid rapid detection with high sensitivity and specificity [19]. Techniques including PCR, nested PCR, and real-time PCR are used for leptospirosis. These can detect *Leptospira*-specific genes such as *ligA*, *ligB*, and *lipL32* genes [44,45]. Real-time PCR can provide diagnostic results immediately after the DNA content of a specific gene is amplified [18]. Nested PCR also aids detection using additional sets of primers for enhanced specificity. While culture is the standard detection method, it requires more time (up to 13 weeks) and a specific medium for growth and the diagnosis of leptospirosis mostly depends on serological tests [25]. This technique detects the specific antibodies produced against the leptospiral antigen through utilizing live bacterial cultures and incubating patient serum with various *Leptospira* serovars [46].

Subgroup analysis of age groups showed that that the pooled prevalence of leptospirosis infection among malaria patients was highest in all age groups (24%), while lower prevalence was demonstrated in studies that enrolled specific groups, such as children (8%) and adults (7%). This result was consistent with the report showing severe leptospirosis occurred more among adolescents than children and adults [47] Moreover, a systematic review showed that 48% of leptospirosis and 42% of deaths were estimated to occur among adult males aged 20–49 years [48]. The meta-analysis also showed that malaria-leptospirosis co-infection was low and that co-infection occurred by chance. The high rate of malaria and leptospirosis co-infection in Bangladesh (75%) might be due to there being innumerable ponds and shallow waters in rural areas of Bangladesh, which facilitate the transmission of the *Leptospira* from rodents to humans [49] or excessive rainfall causing floods facilitating leptospirosis outbreaks in Bangladesh [50]. In Bangladesh, the incidence of malaria cases was reduced and moved in some districts of the country to elimination programs in 2010 [51]. Therefore, the possible explanation of the high rate of malaria and leptospirosis co-infection in Bangladesh might be due to the unavailability of malaria elimination programs during 2007–2010 [39].

The present study had limitations. First, the limited number of studies reported concurrent malaria and leptospirosis infection. Therefore, the limited data, such as clinical laboratory characteristics and also the outcome of coinfected patients that might differ from malaria or leptospirosis mono-infection, could be used to investigate using a meta-analytical approach. Secondly, the pooled prevalence of malaria and leptospirosis co-infection among febrile patients or the pooled prevalence of leptospirosis infection among malaria patients were demonstrated with high prevalence heterogeneity across studies or countries. Therefore, the pooled prevalence of co-infection might not be estimated precisely and should be interpreted with the prevalence of an individual study.

In conclusion, the low prevalence of malaria in leptospirosis co-infection among febrile patients occurred among the included studies. Co-infection was likely to occur by chance. However, clinical symptoms of leptospirosis patients were non-specific and not distinguishable from symptoms of malaria patients. Therefore, clinicians caring for febrile patients in an area where these two diseases are endemic should maintain a high index of suspicion for both diseases, particularly during the peak incidence seasons and whether mono-infections or co-infections are likely. The recognition of co-infection may be an important factor in reducing disease severity and treatment duration.

## Figures and Tables

**Table 1:** Characteristics of the included studies.

Author	Study Site	Year of Conducted	Study Design	Participants	Age	Gender (Male:Female)	Co-Infection	All Malaria Cases	Malaria without Leptospirosis	Leptospirosis without Malaria	Test for Malaria	Test for Leptospirosis
Mueller et al., 2014	Cambodia	2008–2010	Prospective observational studies	1193 febrile patients and 282 non-febrile individuals	7–49 years	801:392	58	676	618	53	Microscopy, RDT, PCR	PCR
Sharma et al., 2014	India	2009	Prospective observational studies	132 febrile patients	≥18 years	NS	8	94	86	4	RDT	Microscopic agglutination test (MAT)
Mehta et al., 2018	India	2012–2013	Prospective observational studies	230 patients with acute kidney injury	≥18 years	NS	2	67	65	30	Microscopy, RDT	ELISA IgM
Wongsrichanalai et al., 2003	Thailand	1999–2002	Prospective observational studies	613 febrile patients	≥20 years	NS	2	18	16	38	Microscopic agglutination test (MAT)	ELISA, microscopic agglutination test (MAT)
Ellis et al., 2006	Thailand	1999–2002	Prospective observational studies	370 febrile patients	20–87 years	325:288	22	155	133	85	Microscopy	ELISA, microscopic agglutination test (MAT)
Swoboda et al., 2014	Bangladesh	2007–2010	Cross-sectional study	659 febrile patients	≥8 years	344:315	30	40	10	96	Microscopy, RDT, PCR	ELISA IgM
Mattar et al., 2017	Colombia	2012–2013	Prospective observational studies	100 febrile patients	1–79 years	62:38	1	4	3	26	Microscopy	ELISA, microscopic agglutination test (MAT)
Raja et al., 2016	India	2013–2014	Cross-sectional study	100 febrile patients	5–60 years	NS	1	10	9	2	Microscopy	ELISA
Lindo et al., 2013	Jamaica	2007–2008	Cross-sectional study	2419 participants testing for dengue	All age groups	1092:1327	10	145	135	147	ELISA	ELISA IgM
Zaki et al., 2010	India	2005	Cross-sectional study	602 febrile patients	1 month to 12 years		3	35	32	24	Microscopy	ELISA IgM
Chipwaza et al., 2015	Tanzania	2013	Cross-sectional study	370 febrile patients	2–13 years	189:191	7	85	78	19	Microscopy	ELISA, microscopic agglutination test (MAT)
Rao et al., 2020	Malaysia	2011–2014	Retrospective observational study	111 leptospirosis-positive patients	Adults	107:4	26	NS	NS	85	Microscopy	PCR, Microscopic agglutination test (MAT)
Singhsilarak et al., 2006	Thailand	NS	Retrospective observational study	194 malaria positive cases	All age	NS	15	194		NS	Microscopy	IFA
Mandage et al., 2020	India	2017–2018	Prospective observational studies	66 malaria positive cases	NS	NS	21	66	61	NS	Microscopy, RDT, PCR	PCR
Forero-Peña et al., 2021	Venezuela	2018	Cross-sectional study	161 patients with *P*. *vivax*	Adults	NS	6	161	NA	NA	Microscopy	ELISA IgM/IgG

## Data Availability

All data related to the present study in this manuscript are available.

## Supplementary Materials

The following are available online at https://www.mdpi.com/article/10.3390/tropicalmed6030122/s1, S1: Prisma 2009 Checklist, Table S1: Search term, Table S2: Quality of the included studies.
