# Supplementary material for: Prevalence of Malaria and Leptospirosis Co-Infection among Febrile Patients: A Systematic Review and Meta-Analysis
Source: Trop Med Infect Dis. 2021 Jul 3;6(3):122. doi: 10.3390/tropicalmed6030122 (PMC8293407; doi:10.3390/tropicalmed6030122)
Supplement: Supplementary file 1 [file tropicalmed-06-00122-s001.zip › Table S1. Search term.pdf]

Prevalence of malaria and leptospirosis co-infection among febrile patients: a systematic review and meta-analysis

Polrat Wilairatana[1], Wanida Mala[2], Wiyada Kwanhian Klangbud[2], Kwuntida Uthaisar Kotepui[2], Pongruj Rattaprasert[3], Manas Kotepui[2*]

[1]Department of Clinical Tropical Medicine, Faculty of Tropical Medicine, Mahidol University, Bangkok, Thailand

[2]Medical Technology, School of Allied Health Sciences, Walailak University, Tha Sala, Nakhon Si Thammarat, Thailand

[3]Department of Protozoa, Faculty of Tropical Medicine, Mahidol University, Bangkok, Thailand

[*]Corresponding author

Manas Kotepui; manas.ko@wu.ac.th, Tel.: +66954392469

Polrat Wilairatana; polrat.wil@mahidol.ac.th

Wanida Mala; wanida.ma@wu.ac.th

Wiyada Kwanhian Klangbud; kwiyada@wu.ac.th

Pongruj Rattaprasert; pongruj.rat@mahidol.ac.th

Kwuntida Uthaisar Kotepui; kwuntida.ut@wu.ac.th

Table S1. Search term

**Databases**	**Search terms**	**Date**
MEDLINE	(Malaria OR plasmodium) AND (Leptospira OR Leptospirosis OR Stuttgart OR Mud OR Rice-Field OR "Rice Field" OR Cane-Cutter OR Canicola)	18 May 2021

	Search results: 148	
Scopus	(Malaria OR plasmodium) AND (Leptospira OR Leptospirosis OR Stuttgart OR Mud OR Rice-Field OR "Rice Field" OR Cane-Cutter OR Canicola)<br><br>Search option: Title, abstract, keywords<br><br>Search results: 1232	18 May 2021
ISI Web of Science	(Malaria OR plasmodium) AND (Leptospira OR Leptospirosis OR Stuttgart OR Mud OR Rice-Field OR "Rice Field" OR Cane-Cutter OR Canicola)<br><br>Search option: All fields<br><br>Search results: 596	18 May 2021
