# Supplementary material for: Prevalence of Malaria and Leptospirosis Co-Infection among Febrile Patients: A Systematic Review and Meta-Analysis
Source: Trop Med Infect Dis. 2021 Jul 3;6(3):122. doi: 10.3390/tropicalmed6030122 (PMC8293407; doi:10.3390/tropicalmed6030122)
Supplement: Supplementary file 1 [file tropicalmed-06-00122-s001.zip › Table S2. Quality of the included studies.pdf]

Prevalence of malaria and leptospirosis co-infection among febrile patients: a systematic review and meta-analysis

Polrat Wilairatana[1], Wanida Mala[2], Wiyada Kwanhian Klangbud[2], Kwuntida Uthaisar Kotepui[2], Pongruj Rattaprasert[3], Manas Kotepui[2*]

[1]Department of Clinical Tropical Medicine, Faculty of Tropical Medicine, Mahidol University, Bangkok, Thailand

[2]Medical Technology, School of Allied Health Sciences, Walailak University, Tha Sala, Nakhon Si Thammarat, Thailand

[3]Department of Protozoa, Faculty of Tropical Medicine, Mahidol University, Bangkok, Thailand

[*]Corresponding author

Manas Kotepui; manas.ko@wu.ac.th, Tel.: +66954392469

Polrat Wilairatana; polrat.wil@mahidol.ac.th

Wanida Mala; wanida.ma@wu.ac.th

Wiyada Kwanhian Klangbud; kwiyada@wu.ac.th

Pongruj Rattaprasert; pongruj.rat@mahidol.ac.th

Kwuntida Uthaisar Kotepui; kwuntida.ut@wu.ac.th

No.	Authors	Eligibility criteria	Study subjects and the setting	Exposure measured in a valid and reliable way 'gold standard'	A specified diagnosis or definition	Confounding factors	Dealing with confounding factors	Outcomes measured in a valid and reliable way	Appropriate statistical analysis	Scores (8)	Quality (high, moderate, low)
1	Chipwaza et al., 2015	Yes	Yes	Yes	Yes	No	NA	Yes	Yes	7	High
2	Ellis et al., 2006	Yes	Yes	Yes	Yes	No	NA	Yes	Yes	7	High
3	Forero-Peña et al., 2021	Yes	Yes	No	Yes	No	NA	Yes	Yes	6	Moderate
4	Lindo et al., 2013	Yes	Yes	No	Yes	No	NA	Yes	Yes	6	Moderate
5	Mandage et al., 2020	Yes	No	Yes	Yes	No	NA	Yes	Yes	6	Moderate
6	Mattar et al., 2017	Yes	Yes	Yes	Yes	No	NA	Yes	Yes	7	High
7	Mehta et al., 2018	Yes	Yes	No	Yes	No	NA	Yes	Yes	6	Moderate
8	Mueller et al., 2014	Yes	Yes	Yes	Yes	No	NA	Yes	Yes	7	High
9	Raja et al., 2016	Yes	Yes	No	Yes	No	NA	Yes	Yes	6	Moderate
10	Rao et al., 2020	Yes	Yes	Yes	Yes	No	NA	Yes	Yes	7	High
11	Sharma et al., 2014	Yes	Yes	No	Yes	No	NA	Yes	Yes	6	Moderate
12	Singhsilarak et al., 2006	Yes	No	Yes	Yes	No	NA	Yes	Yes	6	Moderate
13	Swoboda et al., 2014	Yes	Yes	No	Yes	No	NA	Yes	Yes	6	Moderate
14	Wongsrichanalai et al., 2003	Yes	Yes	Yes	Yes	No	NA	Yes	Yes	7	High
15	Zaki et al., 2010	Yes	Yes	No	Yes	No	NA	Yes	Yes	6	Moderate

NA, Not Applicable
